# Primary Non-refluxing Megaureter: Analysis of Risk Factors for Spontaneous Resolution and Surgical Intervention

**DOI:** 10.3389/fped.2019.00126

**Published:** 2019-04-09

**Authors:** Adriana Dekirmendjian, Luis H. Braga

**Affiliations:** ^1^McMaster Pediatric Surgery Research Collaborative, McMaster University, Hamilton, ON, Canada; ^2^University of Toronto Faculty of Medicine, University of Toronto, Toronto, ON, Canada; ^3^Division of Urology, Department of Surgery, McMaster Children's Hospital, Hamilton, ON, Canada

**Keywords:** primary non-refluxing megaureter, prenatal diagnosis, hydronephrosis, spontaneous resolution, surgical intervention, pediatric

## Abstract

**Background:** The risk of febrile urinary tract infection (fUTI) in children with primary non-refluxing megaureter (PM) has been extensively studied in the literature, however, a paucity of information exists regarding risk factors for surgical intervention and spontaneous resolution. We sought to analyze data from our prospectively collected PM cohort to determine risk factors that would predict surgery and resolution in this population.

**Methods:** Patients with PM were identified from our prospectively-collected prenatal hydronephrosis (HN) database from 2008 to 2017. Primary outcomes included surgical intervention and hydroureter resolution. Spontaneous resolution was defined as ureteral dilation <7 mm at last follow-up. Age at presentation, gender, development of fUTI, HN grade [low (SFU I/II) vs. high (SFU III/IV)], anteroposterior diameter (APD) measurements and ureteral diameter at baseline and last follow-up were recorded. Univariate and multivariable analyses (binary logistic and Cox regression) were performed.

**Results:** Of 101 patients, 86 (85%) were male, and 80 (79%) had high grade HN. Median age at baseline and last follow-up were 2 (0–23) and 29 (2–107) months, respectively. Overall, 23 (23%) patients underwent surgery at a median age of 22 (3–35) months. Mean ureteral diameter was larger in surgical patients vs. those treated non-surgically (14 ± 4 vs.11 ± 3 mm; *p* < 0.01). Of the 78 (77%) non-surgical patients, 43(55%) showed resolution of their ureteral dilation at a median age of 24(4–56) months. Survival analysis demonstrated that 12 patients resolved by year 1, 22 by year 2, 30 by year 3, 40 by year 4, and 43 by year 5. However, when considering resolution as APD <10 mm, 62(79%) children resolved their HN by last follow-up (29 months). Univariate and multivariable analyses (Table 1) revealed that high-grade HN at baseline, development of fUTI, and ureteric dilation ≥14 mm were significant risk factors for surgical intervention. Cox regression (Figure 2) found that ureteral dilation <11 mm was the only independent risk factor significantly associated with PM resolution (Table 2).

**Conclusion:** Patients with PM and high-grade HN, as well as individuals with ureteral dilation ≥14 mm and fUTI were more likely to undergo surgical intervention. Ureteral dilation <11 mm was the only independent risk factor significantly associated with spontaneous resolution of PM.

## Introduction

Primary non-refluxing megaureter (PM) is a congenital dilatation of the ureter, primarily affecting males, which accounts for 5–10% of all prenatal hydronephrosis (HN) cases (1–4). Previously, the majority of children with PM did not present to pediatric nephrologists and urologists until the development of symptoms such as febrile urinary tract infection (fUTI), hematuria and flank/abdominal pain. Subsequently, most of these children underwent surgical intervention in order to alleviate symptoms and manage ureteric dilatation (3–5). Since the widespread use of fetal ultrasonography, urologists have been able to increasingly detect PM prenatally and have observed that in the post-natal period, many of these cases remain asymptomatic and clinically stable, thereby allowing for conservative management (4, 5). As a result, many children with PM have avoided surgical intervention and physicians have increasingly adopted an initial watchful waiting approach (5, 6). Additionally, several studies have demonstrated spontaneous resolution of prenatally diagnosed PM, further supporting non-operative management (1, 3–6).

Not all cases, however, are effectively controlled with conservative management alone, and many physicians recommend some form of surgical intervention if patients become symptomatic, renal function becomes decreased or spontaneous resolution is not achieved after a few years of observation (3, 4). A paucity of information still exists, however, with regards to which children benefit significantly from surgical intervention and what additional clinical criteria should be used as predictive factors for surgery. Elucidating such criteria could therefore assist physicians in determining, which children should be more closely monitored for future surgical intervention. Although non-operative management has been shown to be safe and effective, a lack of consensus and data persists as to which characteristics suggest a higher likelihood of spontaneous resolution without intervention, specifically regarding distal ureteric diameter (4–6). The focus of this study is therefore to analyze a prospectively collected cohort of children diagnosed with prenatal HN, specifically PM, in order to determine factors predictive for surgery and spontaneous resolution, as well as time to spontaneous resolution in this population of patients (7, 8). We chose not to address the development of fUTI in this cohort, as the senior author previously published a study, which examined risk factors for fUTI in PM patients extensively (1).

## Materials and Methods

### Setting, Population and Study Inclusion and Exclusion Criteria

After receiving approval from the Hamilton Integrated Research Ethics Board (REB 15-162-D), our local ethics board, we reviewed the records of patients captured in our prospectively collected prenatal HN database, which includes all patients referred to a tertiary pediatric hospital for prenatal HN from 2008 to 2017. We excluded all patients older than 24 months and those without diagnosed HN, as confirmed by postnatal renal ultrasound. Furthermore, patients with diagnosed vesicoureteral reflux (VUR), duplicated collecting systems, ureterocele, ectopic ureter, posterior urethral valves, neurogenic bladder/spina bifida, or prune belly were excluded. As a result, the remaining 101 infants with PM were included in our study population and subsequent analysis.

PM was defined as HN with ureteral dilation ≥ 7 mm, which was confirmed on postnatal ultrasound and absent VUR as documented by voiding cystourethrogram. This definition is in accordance with the Campbell-Walsh textbook, which states that megaureter is a descriptive term that denotes dilatation of the ureter that is 7 mm or greater, irrespective of cause (9). The indications for voiding cystourethrogram in prenatal HN cases were based on physician discretion and included high grade HN (SFU III/IV), ureteral dilation and bladder abnormalities on ultrasound (large capacity and thickening of the wall). Our indications for surgery were in keeping with those described by Wein et al. who state that surgery should be considered in the setting of symptoms or recurrent UTIs, progressive unremitting dilatation on ultrasound, differential renal function <40% and/or significant decreases in differential renal function of 5% or great on renal nuclear functional studies (9). Based on the institutional protocol, all patients with PM and high grade HN were initiated on continuous antibiotic prophylaxis (CAP) from birth until 12 months of age, except for 33 children whose CAP status was unknown due to concomitant enrollment in a randomized controlled trial (10).

### Primary Outcome of Interest and Independent Variables

**Figure 1:**
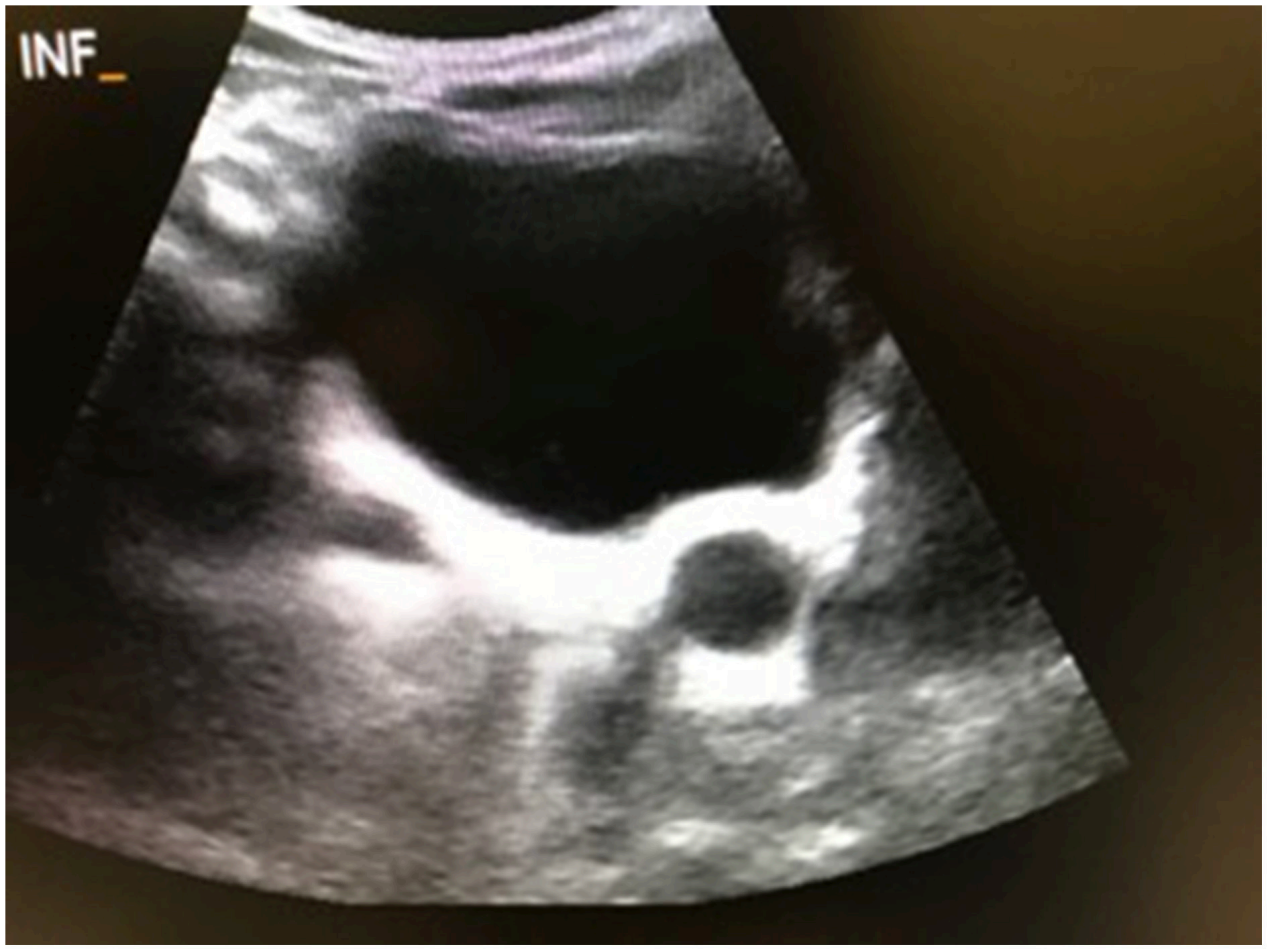
Transverse ultrasound view of bladder with dilated ureter.

The primary outcomes in this study were spontaneous resolution of PM, which was defined as a distal ureteral dilation <7 mm at last follow-up, as well as surgical intervention, which included ureteral reimplantation (± tapering), distal side-to-side refluxing ureterocystostomy, and distal cutaneous ureterostomy. Potential risk factors for surgical intervention and spontaneous resolution were chosen a priori and included gender, development of fUTI, HN grade (low vs. high), ureteral dilation (<14 vs. ≥14 mm and <11 vs. ≥ 11 mm), and renal function, as determined by MAG3 lasix renal scintigraphy. Ureteral dilation cut-offs were derived from the senior author's previous study (1). Patients who underwent surgery were included in the analysis of resolution only until the time of surgery, at which point they were censored. Low grade HN was defined as SFU grade I/ II, and high grade HN was defined as SFU grade III/IV. All patients with SFU grade IV HN had evidence of parenchymal thinning on ultrasound. Development of fUTI was defined by documented fever (>38°C), pyuria (urinalysis with positive leukocyte esterase and >10 white blood cells per high power field), bacteria on Gram stain and urine culture yielding >100,000 cfu/mL of a single microorganism from a catheterized specimen. Ureteral dilation was determined based upon the largest ureteral caliber measured at its most distal part on transverse ultrasound view of the bladder (Figure 1).

### Statistical Analyses

Univariate analyses were performed on the five a priori variables for surgical intervention and spontaneous resolution, using Chi-square and Fisher's exact tests. Student's sample *t*-tests were used to compare continuous variables between groups. Furthermore, multivariable analyses using binary logistic and Cox regression models were utilized, adjusting for time dependent variables that effect spontaneous resolution rates. Hazard ratios with the 95% CI were calculated for each predictor variable. Kaplan-Meier curves were applied to illustrate the cumulative rate of resolved and unresolved HN as a measure of time. All statistical analyses were done with SPSS, version 22 with p<0.05 considered statistically significant.

## Results

The study population consisted of 101 patients with PM, including 86 (85%) males, 30 (35%) of whom had undergone circumcision for religious reasons or parental preference, and 15 (15%) females. Median age at baseline, which was defined as the first clinic visit, was 2 months (range 0–23) and the median age at last follow-up was 29 months (range 2–107). In this study population 21 (21%) individuals had low grade HN (SFU I/II), while 80 (79%) had high grade HN (SFU III/IV). 76 (75%) patients underwent renal scintigraphy, and the mean baseline differential renal function (DRF) was 52 ± 14%. When examining ureteral dilation, we found that the mean ureteral dilation at baseline was 12 ± 4 mm, 30 (30%) patients had ureteral tortuosity, and 24 (24%) patients developed fUTI (Table 1). Of the patients with fUTI, 22 (92%) were male, 17 (71%) had high grade HN, the median age at the time of development of fUTI was 5 months (range 1–31), and the mean ureteral dilation was 13 ± 4 mm. Fifteen (63%) patients were receiving CAP at the time of fUTI and 9 (27%) patients who developed fUTI had an unknown CAP status.

**Table 1:** Patient Characteristics.

**Patient Characteristics**	***n* = 101 (%)**
**GENDER**	
Male	86 (85)
Female	15 (15)
**HN GRADE**	
Low grade (SFU I/II)	21 (21)
High grade (SFU III/IV)	80 (79)
Median age at baseline (mos.) (min-max)	2 (0–23)
Median age at last follow up (mos.) (min-max)	29 (2–107)
Mean ureteral dilation at baseline ± SD	12 ± 4
**fUTI**	
Yes	24 (24)
**RENAL FUNCTION (*****n*** **= 76)**	
<40%	9 (9)
≥40%	67 (66)
Mean renal function at baseline (%) ± SD	52 ± 14
Mean t ½ at baseline (mins.) ± SD (*n* = 71)	27 ± 54

Analysis established that 23 (23%) patients underwent surgical intervention, with 13 (57%) children receiving ureteral reimplantation (± tapering). The median age at the time of surgery was 22 months (range 3–32) and the mean ureteral dilation ± SD was 17 ± 5 mm. Additionally, 8 (35%) patients who underwent surgery had a t ½ time of >30 min on MAG3 lasix renal scan. Univariate analysis was performed to identify whether any of the a priori variables were significantly associated with surgical intervention. This analysis demonstrated that infants with high-grade HN had a significantly higher rate of surgical intervention compared to those with low-grade HN (28 vs. 5%, *p* = 0.04). Similarly, development of fUTI was a significant predictor of surgery (46 vs. 16%, *p* < 0.01), as well as ureteral dilation, with 48% of surgical patients presenting with ureteral dilation ≥14 mm, in comparison to the 15% with ureteral dilation <14 mm (*p* < 0.01). Contrastingly, DRF was not a significant predictor of surgery, as only 3 (33%) patients with renal function <40% underwent surgery vs. 20 (30%) patients with renal function ≥40% who were managed surgically (Table 2). After controlling for confounding variables using a Cox regression model, we observed that ureteral dilation ≥ 14 mm (HR 7.8, 95% CI 2.0–31.0, *p* < 0.01) and development of fUTI (HR 8.8, 95% CI 2.2–35.3, *p* < 0.01) were the two independent variables significantly associated with surgical intervention (Table 2).

**Table 2:** Univariate and multivariable analysis of risk factors for surgical intervention.

		**Univariate**		**Multivariable**	
	**Surgery *n* = 23 (%)**	**Total *n* = 101**	***p*-value**	**HR (95% CI)**	***p*-value**
**GENDER**					
Male	20 (23)	86	1.00	Ref	0.67
Female	3 (20)	15		1.4 (0.3–7.2)	
**HN GRADE**					
Low grade (I/II)	1 (5)	21	0.04	Ref	0.24
High grade (III/IV)	22 (28)	80		4.8 (0.3–66.0)	
**fUTI**					
Yes	11 (46)	24	<0.01	Ref	<0.01
No	12 (16)	77		8.8 (2.2–35.3)	
**URETERAL DILATION**					
<14 mm	12 (15)	78	<0.01	Ref	<0.01
≥14 mm	11 (48)	23		7.8 (2.0–31.0)	
**RENAL FUNCTION**					
<40%	3 (33)	9	1.00	Ref	0.08
≥40%	20 (30)	67		5.0 (0.8–30.1)	

**Figure 2:**
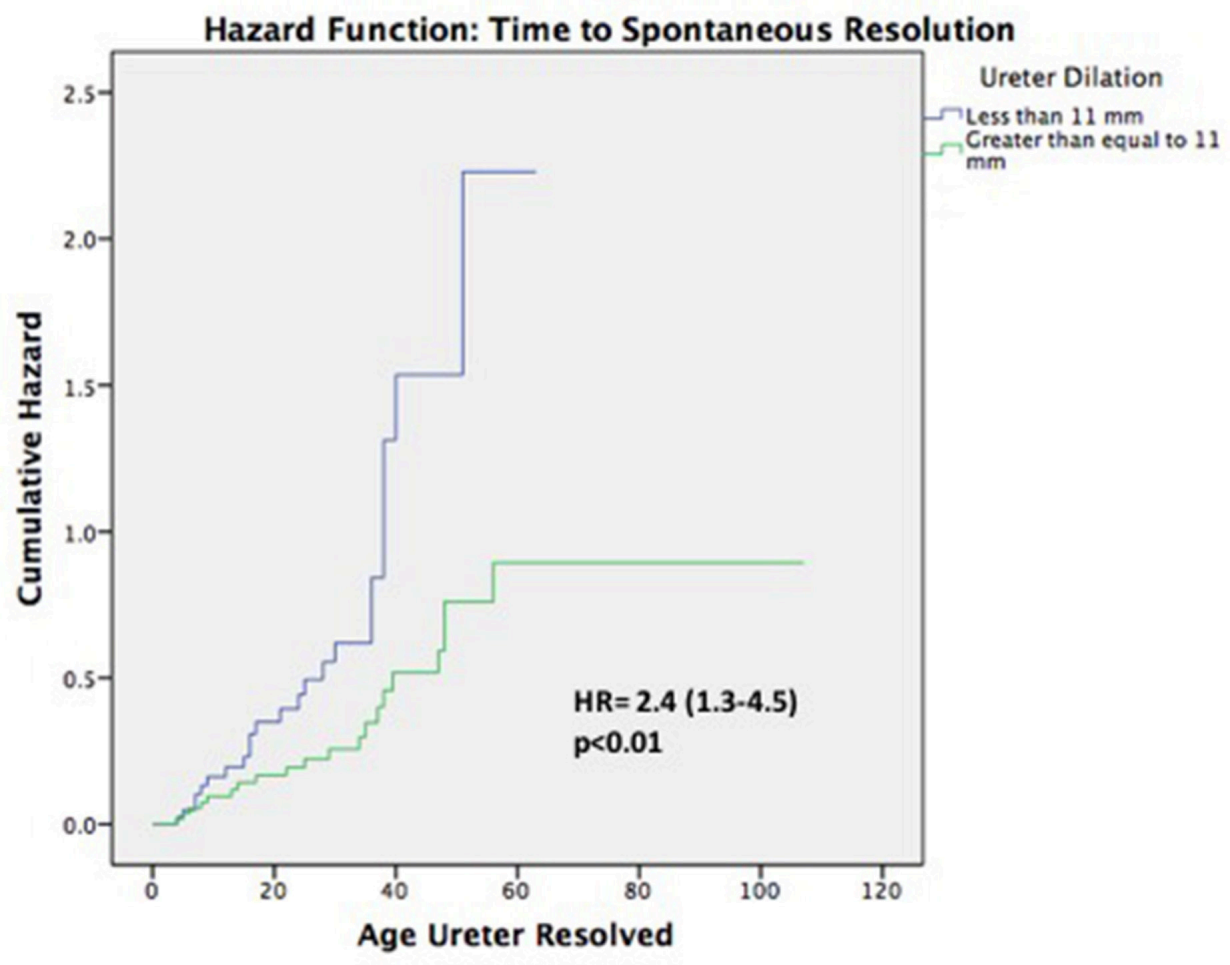
Cox proportional regression analysis of ureteral dilation.

Of the remaining 78 (77%) non-surgical patients, 43 (55%) demonstrated spontaneous resolution of ureteral dilation at last follow-up. The median age at resolution was 24 months (range 4–56) and survival analyses demonstrated that 12 (15%) patients resolved by year 1, 22 (28%) resolved by year 2, 30 (38%) resolved by year 3, 40 (51%) resolved by year 4, and 43 (55%) resolved by year 5 (Figure 2). Mean ureteral dilation at baseline was 11 ± 4 mm, and at the time of resolution mean ureteral dilation and mean anteroposterior diameter (APD) were 1 ± 2 mm and 5 ± 3 mm, respectively. On univariate analysis, HN grade was the only factor significantly associated with spontaneous resolution, with 62 and 38% of resolved patients diagnosed with low and high grade HN at baseline, respectively (*p* = 0.04), however Cox proportional regression analysis demonstrated that patients with ureteral dilation of <11 mm were 2.4 times more likely to resolve spontaneously than those with a ureteral diameter ≥ 11 mm (HR 2.4, 95% CI 1.3–4.5, *p* < 0.01) (Table 3).

**Table 3:** Univariate and multivariable analysis of risk factors for spontaneous resolution.

		**Univariate**		**Multivariable**	
	**Resolution *n* = 43(%)**	**Total *n* = 101**	***p*-value**	**HR (95% CI)**	***p*-value**
**GENDER**					
Male	36 (42)	86	0.73	Ref	0.69
Female	7 (47)	15		1.2 (0.5–2.7)	
**HN GRADE**					
Low grade (I/II)	13 (62)	21	0.04	Ref	0.19
High grade (III/IV)	30 (38)	80		1.6 (0.8–3.0)	
**URETERAL DILATION**					
<11 mm	23 (53)	43	0.06	Ref	<0.01
≥11 mm	20 (34)	58		2.4 (1.3–4.5)	

## Discussion

The transition from surgical to non-surgical initial management in the treatment of children with PM has been well-documented, and a non-operative approach has been shown to be safe and effective for the majority of prenatally-diagnosed PM patients (5, 6, 11–13). Although studies have documented high rates of resolution, indicators for surgical intervention remain controversial (14).

We observed that the majority of patients in our study were prenatally diagnosed male infants, which is consistent with previous studies (14, 15). The median age at baseline/diagnosis in our patient group was 2 months, which supports the findings reported by Rubenwolf et al. (14) who found that the age of diagnosis was significantly lower in children diagnosed between 2005 and 2009 (4.1 months) in comparison to those diagnosed between 1986 and 1990 (25.3 months). This decreasing trend in age at diagnosis reflects the impact of fetal ultrasonography and the changing management paradigm of PM, whereby children are being increasingly diagnosed on prenatal ultrasound rather than after becoming symptomatic at a later age (11, 14). A younger age at diagnosis has the potential to substantially impact patients, as we are able to identify early those at increased risk for fUTIs, and begin CAP before the development of such illnesses. Conversely, a rigorous monitoring protocol adds stress to families, cost to the health care system and exposure to medications and radiation to children who are likely to improve on their own. Clearly, better selection and risk stratification would help with management.

It has been previously demonstrated that children with PM are at an increased risk for developing fUTI. In their study of 44 patients with PM, Ranawaka and Hennayake (3) observed fUTI in 16% of patients, with a higher proportion of fUTIs occurring in children with ureteral dilation ≥10 mm. Similarly, a previous study by Braga et al. (1) reported a 34% fUTI rate occurring at a mean age of 5.8 months, with 82% of infections occurring in children with high grade HN. Lee et al. (16) also demonstrated development of fUTI in 47% of children from a subset of PM patients, and reported that the vast majority of infections occurred before 6 months of age. Our study showed comparable findings, with an incidence of fUTI in 24% of patients at a median age of 5 months. We also observed that the proportion of infections was substantially higher in males with high grade HN (92 and 71%, respectively). The slightly lower incidence of fUTIs observed in our cohort in comparison to that seen by Lee et al. (16) and Rubenwolf et al.'s (14) (43% UTI rate) may be explained by the fact that our institutional practice is to recommend CAP to infants with high grade (III/IV) HN. As a result, the majority of patients included in our study population were either receiving or had received CAP for prevention of fUTI. Based on this mounting evidence, in conjunction with our findings, we continue to recommend that children with high grade PM are maintained on CAP, especially up to 6 months of age, in order to prevent the development of fUTI or promptly detect breakthrough infections in a timely manner in order to avoid potentially damaging sequelae, as well as hospitalization (4, 14, 15, 17). We await the results of a randomized controlled study on the topic to provide more conclusive evidence supporting or disputing this practice.

Various long-term studies have demonstrated the potential for PM to resolve spontaneously over time (1, 4–6, 12–15, 18). Similarly, our findings demonstrate that 55% of non-surgically managed patients experienced spontaneous resolution at a median age of 24 months. This figure is well within and consistent with previously reported rates ranging between 39 and 85%. This resolution rate is also supported by a retrospective study of 212 children with PM, which demonstrated that 39% of non-surgically managed children reached resolution (14). Contrastingly, in a previous study performed by Braga et al. (1), it was shown that 85% of patients who did not require surgery experienced spontaneous resolution during a median follow up period of 19 months. Although this resolution rate is higher than our observed 55%, it is important to note that in the former, study resolution was defined as APD ≤10 mm, ureteral dilation <8 mm and/or SFU grade II or less by last follow up, thereby broadening the criteria for spontaneous resolution.

Despite variance in resolution rates, it is widely accepted that non-surgical management of asymptomatic PM is highly effective and safe, however the predictive factors of resolution are still controversial and not well-established. Our analysis demonstrated that ureteral dilation <11 mm at baseline was the only significant independent variable associated with spontaneous resolution. This finding is similar to that of other studies, which have reported a higher incidence of spontaneous resolution in children with ureteral dilation ranging from 8 to 13 mm (3–6, 19). After multivariable analysis we no longer found hydronephrosis grade to be significantly associated with spontaneous resolution. Calisti (12), DiRenzo (18), and Gimpel (4) similarly found a weak/absent correlation between grade, ureteral dilation and average time to resolution. Conversely Chertin (19), McLellan (5), and Arena (6) concluded that hydronephrosis grade was predictive of resolution rate and found a positive correlation between age at resolution and increasing severity of HN. We hypothesize that HN grade is not truly predictive of resolution, as it has been observed that substantial ureteral dilation may occur even in the context of mild renal pelvis dilatation (4). Rather, this variable appears to be a confounding factor, as indicated by its lack of significance in multivariable models. As a result, we propose that clinicians base their estimations for resolution primarily upon ureteral dilation at baseline, instead of relying upon the grade of HN.

Of those patients that did not achieve spontaneous resolution in our cohort, 66% either remained stable or experienced reduction in ureteral dilation by the last reported follow-up. Furthermore, the median age at follow-up in our unresolved subgroup was 21 months, thereby indicating that resolution at a later age is plausible given the trends we observed, with patients demonstrating resolution of ureteral dilation as late as 56 months of age. Similarly, Ranawaka and Hennayake (3) and Gimpel et al. (4) found that a median time of 5 and 7 years, respectively, were required to reach resolution. In one of the few long-term studies on the topic Shukla et al. (13) demonstrated that although non-surgical management was an effective approach for patients who remained unresolved, there was the potential for complications to arise as late as 14 years due to residual ureteral dilation. We therefore, recommend that consistent, non-surgical management should be continued until dilation resolves (ureteric diameter <7 mm), a treatment approach which has been supported by many previous authors as well (4–6, 12–15, 18).

As reported by DiRenzo (18), and Gimpel (4) almost a quarter of patients in our study population underwent surgical intervention in the form of ureteral reimplantation (± tapering), distal side- to-side refluxing ureterocystostomy or distal cutaneous ureterostomy. Our main surgical indications included delayed drainage, worsening HN on repeat US and fUTI, which is consistent with protocols reported by others (1, 3, 5, 20). Although prior studies have proposed that a ureteral diameter > 10 mm be considered an indicator for persistent megaureter and the need for surgery, the results of our study suggest otherwise, as 53% of children in this study with a ureteral diameter <11 mm achieved resolution of dilatation with conservative management alone (5, 12, 21). Similar to results by Chertin (19) and Calisti (12) our study demonstrates that a ureteral diameter of 14 mm should be considered a significant risk factor for surgical intervention, as 48% of children in this subgroup required operative management. Various authors have also considered DRF <40% at baseline as a frequent indication for surgery in patients with PM (14, 20, 22). However, only 3 infants in our series with a DRF <40% underwent surgical intervention, which did not reach statistical significance on univariate and multivariable analyses. Furthermore, in their study of 25 patients (DRF ranging from 10 to 40%) who were allocated to receive either conservative or surgical management, Drlik et al. (11) observed no significant differences between the groups and noted a similar increase of DRF in both groups. They thereby concluded that an initial, low DRF in an asymptomatic, stable patient should not be an independent indication for surgery.

Our findings should be considered in the context of the limitations of the study. The reported study population is comprised of a rather small number of patients from a single center, which may potentially limit the generalizability of our results. Furthermore, the study results demonstrate a limited number of events with regards to surgical intervention and spontaneous resolution and as such we were unable to include multiple variables in our multivariable analysis, as the ideal model has an outcome to variable ratio of approximately 10:1; the robustness of our model was thereby restricted by our number of outcomes, specifically with regards to spontaneous resolution. However, in their paper on logistic and Cox regression, Vittinghoff and McCullough (23) demonstrated that an outcome to variable ratio ranging from 5 to 9:1 is still statistically reliable. Although indications for surgery were objective and consistent with those described in previous studies, the decision to operate ultimately resided with a single surgeon, rather than being based upon an institution protocol, thus introducing, potential bias. Lastly, our median follow-up of 29 months may not be long enough to detect some of the reported long-term complications, which may develop as late as 14 years after infancy. Despite these limitations, we believe there is still substantial value in our study due to its prospectively collected data, strict criteria for inclusion and outcome analysis, and its further insight into predictive factors for surgery and spontaneous resolution.

## Conclusion

Observational management remains the mainstay initial strategy for management of asymptomatic, prenatally diagnosed PM. Our study adds to the growing body of evidence which suggests that a watchful waiting approach is safe and effective, as the majority of patients will experience complete spontaneous resolution, reduction of ureteral dilation, or remain asymptomatic with stable dilation. When prognosticating PM patients with regards to spontaneous resolution, our findings suggest that the only independent, predictive variable that should be considered is ureteral dilation <11 mm at baseline, as these patients are more likely to resolve within 24 months of age and may benefit from less stringent monitoring protocols. Patients with ureteral dilation ≥ 14 mm, and those who develop fUTI, are significantly more likely to undergo surgical intervention and should be monitored closely.

## Author Contributions

LB and AD were responsible for the design of the study, acquisition, analysis and interpretation of data, drafting the manuscript, revising the manuscript critically for important content and approval of the final version of the manuscript.

### Conflict of Interest Statement

The authors declare that the research was conducted in the absence of any commercial or financial relationships that could be construed as a potential conflict of interest.
